# Diagnostic and therapeutic value of progastrin‐releasing peptide on small‐cell lung cancer: A Single‐Center Experience in China

**DOI:** 10.1111/jcmm.13722

**Published:** 2018-07-10

**Authors:** Xiao‐Yuan Wu, Yang‐Bo Hu, Hui‐Juan Li, Bing Wan, Chen‐Xi Zhang, Bin Zhang, Huan Hu, Qun Zhang, Tang‐Feng Lv, Ping Zhan, Yong Song

**Affiliations:** ^1^ Central Laboratory Nanjing Chest Hospital School of Medicine Southeast University Nanjing China; ^2^ Department of Respiratory Medicine Jinling Hospital Medical School of Southeast University Nanjing China; ^3^ Department of Respiratory Medicine Jinling Hospital Nanjing University Institute of Respiratory Medicine Nanjing University School of Medicine Nanjing China; ^4^ Department of Respiratory and Critical Medicine Nanjing Jiangning Hospital The Affiliated Hospital of Nanjing Medical University Nanjing China; ^5^ Department of ICU The Affiliated Hospital of Jiangsu University Zhenjiang China; ^6^ Department of Respiratory Medicine and Central Laboratory Nanjing Chest Hospital School of Medicine Southeast University Nanjing China

**Keywords:** diagnosis, neuron‐specific enolase, progastrin‐releasing peptide, small‐cell lung cancer, therapeutic monitoring

## Abstract

We aimed to compare the diagnostic efficiency of proGRP and NSE on SCLC and to investigate whether the change of proGRP level would predict therapeutic response. Patients who were firstly diagnosed pathologically in Nanjing Chest Hospital and measured proGRP level consecutively were enrolled in the study. ProGRP level was detected using Elecsys ProGRP Assay. Totally 75 SCLC, 234 NSCLC and 264 benign lung diseases (BLD) were enrolled. Both proGRP and NSE levels in SCLC were significantly higher than those in NSCLC and BLD, and proGRP in extensive stage SCLC was higher than which in limited stage (*P* ≤ .001). The diagnostic efficiency of proGRP on SCLC was higher than that of NSE, but when the two biomarkers were bind together, the diagnostic efficiency was the best. When SCLC was differentiated from NSCLC and BLD, the cut‐off values were 114.35 pg/mL and 162.55 pg/mL respectively. For treatment responsive patients, proGRP level decreased markedly after the first cycle of therapy and kept a continued momentum of decline during treatment. But for unresponsive patients, no obvious decline was observed. ProGRP had higher diagnostic efficiency on SCLC when compared to NSE, and it could better predict therapeutic response of pulmonary target lesions on chemotherapy.

## 1 INTRODUCTION

Lung cancer is among the top in incidence of malignant cancers and leads to the highest mortality rate in China, either male or female.^1^ Although only 13% of lung cancers patients were diagnosed with small‐cell lung cancer (SCLC), more than half of which were extensive stages.^2^, ^3^ However, when compared to NSCLC, treatment of SCLC remains stagnant. Despite of its sensitivity to chemotherapy and radiotherapy, the 5‐year survival was <7%,^4^ because of early metastasis to distant organs and lymph nodes and high relapse rate. Thus, diagnosis at early stage and periodical monitoring was crucial for improving survival of SCLC patients.

Gastrin releasing peptide (GRP) was firstly isolated from porcine stomach in 1978 and then it was found to be present in many other organs and tissues, such as foetal lung and lung cancers, especially SCLC.^5^, ^6^, ^7^, ^8^ In 1988, the possibility of GRP being a biomarker of SCLC was aware.^9^ Since then, a quantity of studies explored GRP as a biomarker of SCLC but found it challenge because GRP is unstable and it is hard to measure its level in plasma. Progastrin‐releasing peptide (proGRP) is the precursor of GRP, and it was more stable in plasma. Consequently, assays that measure the level of proGRP were developed,^10^ and several studies showed it was an effective biomarker of SCLC with high sensitivity and specificity.^11^, ^12^

Neuron‐specific enolase (NSE) was the preferred tumour marker of SCLC in the early days. However, some studies demonstrated that the diagnostic sensitivity of NSE on SCLC was lower than that of proGRP when the specificity was fixed at 95%.^13^, ^14^ Even though, the sensitivity of proGRP in diagnosing SCLC was inconsistent in various studies. Particularly, the cut‐off value of proGRP was variant in clinic. For example, Yang et al^15^ found the sensitivity and specificity of 75% and 100%, respectively, at the cut‐off value of 300 pg/mL; Oh et al^16^ showed 85.7% sensitivity and 90.2% specificity at cut‐off value of 63 pg/mL; Nisman et al^17^ demonstrated a new assay of plasma proGRP distinguishing SCLC from NSCLC with 80.4% sensitivity and 96.3% specificity at cut‐off value of 140 pg/mL. Inappropriate cut‐off values may bring about under‐diagnosis and misdiagnosis.

In this study, we retrospectively collected patients who were firstly diagnosed at Nanjing Chest Hospital without any treatment and measured proGRP and NSE levels before and during follow‐up of treatment. We aimed to analyse and compare the diagnostic efficiency of proGRP and NSE on SCLC and to investigate whether the change of proGRP level would predict therapeutic response.

## 2 MATERIALS AND METHODS

### 2.1 Patients

We searched through patients records in Nanjing Chest Hospital and patients meeting the following criteria were included: (i) firstly diagnosed without any treatment; (ii) diagnosis was pathologically confirmed; (iii) proGRP and NSE levels before treatment were measured; (iv) detecting proGRP levels in succession during follow‐up of treatment; (v) renal function indicators such as serum creatinine, blood urine nitrogen and creatinine clearance rate were within normal limits. Data including age, sex, pathological diagnosis, stages, proGRP and NSE levels before treatment, proGRP levels in succession during treatment, treatment regimens and therapeutic responses were gathered. The therapeutic response was evaluated according to Response Evaluation Criteria in Solid Tumors (version 1.1),^18^ and it was class to two groups: responders and non‐responders. Responders including complete remission (CR) and partial remission (PR), and non‐responders consisted of stable disease (SD) and progressive disease (PD). This study was approved by the Study Ethics Committee of Nanjing Chest Hospital, and the application of exemption from written informed consent was allowed because of a retrospective design.

### 2.2 ProGRP assay

The Elecsys ProGRP assay, an electrochemiluminescence immunoassay, is based on the principle of double antibodies sandwich method, in which biotinylated and ruthenium‐labelled ProGRP‐specific mouse monoclonal antibodies are used to capture and detect ProGRP. Both plasma and serum specimens were permissible for detection in the Elecsys ProGRP assay because the antibody combination sites avoid the protease cleavage area, so that the proGRP would be degraded by the endogenous protease formed during the agglutinating process in serum.

During the first incubation, the specimen (30 μL) and the biotinylated and ruthenium‐labelled ProGRP‐specific mouse monoclonal antibodies formed sandwich complexes; then, streptavidin coating magnetic beads were added and the double‐antibody sandwich complexes bound to these magnetic beads by the interaction of biotin and streptomycin. Afterwards, the reaction media was pipetted to the measuring cell, and magnetic beads were absorbed on electrodes; uncombined substance was removed by ProCell (No. 11662988122)/ ProCell M (No. 04880340190). Giving specific voltage to electrode and the complexes would give out light, and the light intensity could be measured by photomultiplier. Finally, results would be obtained according to the calibration curve.

### 2.3 Statistical analysis

Skewed distributed data were presented as median and quartile (Q, 25th and 75th percentiles), and statistical analysis was performed using non‐parametric test. Comparison of multiple groups of random samples was analysed by Kruskal‐wallis test. Data following normal distribution, such as age, were expressed as mean and standard deviation (SD). Receiver operating characteristic (ROC) curves could visually display the correlation between sensitivity and specificity, and areas under curve (AUC) was calculated to assess the diagnostic efficiency. Change of proGRP levels following treatment was performed using Wilcoxon signed‐rank test. All the statistical analysis was performed using SPSS version 20.0 (IBM Co.; Armonk, NY, USA) and GraphPad Prism version 6.01 (GraphPad software Inc.; La Jolla, CA, USA). *P* < .05 was considered as significant.

## 3 RESULTS

### 3.1 Patients characteristics

**Figure 1:**
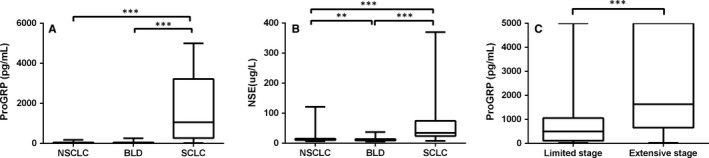
ProGRP and NSE level in SCLC, NSCLC and BLD. A, proGRP level in SCLC, NSCLC and BLD; B, NSE level in SCLC, NSCLC and BLD; C, proGRP level in limited and extensive stages SCLC. proGRP, progastrin releasing peptide; SCLC, small‐cell lung cancer; NSCLC, non‐small‐cell lung cancer; BLD, benign lung diseases. **P < 0.01, ***P < 0.001

Totally, 573 patients were enrolled, and among them, 68.0% were male and 32.0% were female. The average age was 61.9 ± 13.8 (mean ± SD) years old. Patients were divided into three groups according to histopathological characteristics: SCLC, NSCLC and benign lung diseases (BLD). BLD mainly consisted of pulmonary infection, bronchiectasis and chronic obstructive pulmonary diseases (COPD), et al. Among them, 75 were SCLC, 234 were NSCLC and 264 were BLD. The median concentration of proGRP level of SCLC patients was 1058.00 pg/mL (Q, 268.20‐3218.25 pg/mL), which was significantly higher than that in NSCLC (median 37.46 pg/mL, 29.61‐49.87 pg/mL) and BLD (median 37.08 pg/mL, 26.74‐54.11 pg/mL) (both *P* < .001; Figure 1A). But no difference was noticed between NSCLC and BLD. The median level of NSE was 35.06 pg/mL (24.13‐73.43 pg/mL) in SCLC patients, 12.26 pg/mL (10.42‐15.57 pg/mL) in NSCLC patients and 11.14 pg/mL (9.44‐13.79 pg/mL) in benign lung diseases. And NSE levels of all the three groups were significantly different from each other (*P* ≤ .001; Figure 1B).

For SCLC patients with identified stages, 16 were limited stages and 40 were extensive stages. The median proGRP level of limited stage patients was 660.20 pg/mL (88.05‐1674.00 pg/mL), which was markedly lower than that of extensive stages (1632.50 pg/mL, 657.80‐5000.00 pg/mL; Figure 1C).

### 3.2 Diagnostic efficiency of proGRP and NSE

To determine the diagnostic efficiency of proGRP and NSE on SCLC, ROC curves were performed. However, when distincting SCLC from different diseases, distinct cut‐off values of proGRP were identified. Therefore, ROC curves differentiating SCLC from NSCLC and BLD were performed, respectively.

#### 3.2.1 SCLC vs NSCLC

**Figure 2:**
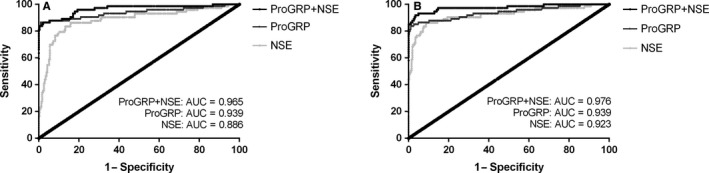
Receiver operating characteristic (ROC) curves of proGRP, NSE, and their combination on the diagnosis of SCLC. A, SCLC vs NSCLC; B, SCLC vs BLD. ROC, receiver operating curve; proGRP, progastrin releasing peptide; NSE, neuron‐specific enolase; SCLC, small‐cell lung cancer; NSCLC, non‐small‐cell lung cancer; BLD, benign lung diseases; AUC, area under curve

When discriminating SCLC from NSCLC, the cut‐off value of proGRP was 114.35 pg/mL and of NSE was 17.34 μg/L (where the Youden Index was maximal); both the sensitivity was 86.5%, while the specificity of proGRP was 99.1% and of NSE was 84.2%; AUC of proGRP was 0.939, which was higher than that of NSE (0.886). (Figure 2A). However, the cut‐off value used clinically in our hospital was 65.7 pg/mL, where the sensitivity was 87.8% and specificity was 91.5%. (Table 1).

**Table 1:** Diagnostic value of proGRP, NSE and their combination on SCLC

Diseases	Biomarkers	Cutoff value	Sensitivity (%)	Specificity (%)	AUC
SCLC vs NSCLC	proGRP	114.35 pg/mL	86.5	99.1	0.939
		65.7 pg/mL	87.8	91.5	‐
	NSE	17.34 μg/L	86.5	84.2	0.886
	proGRP + NSE	‐	86.5	97.9	0.965
SCLC vs BLD	proGRP	162.55 pg/mL	83.8	98.9	0.939
		65.7 pg/mL	87.8	86.7	‐
	NSE	17.35 μg/L	86.5	91.3	0.923
	proGRP + NSE	‐	91.9	96.6	0.976

AUC, area under curve; BLD, benign lung diseases; NSCLC, non‐small‐cell lung cancer; ‐, no information; NSE, neuron‐specific enolase; proGRP, progastrin releasing peptide; SCLC, small‐cell lung cancer

#### 3.2.2 SCLC vs BLD

However, for SCLC and BLD, the cut‐off value of proGRP at maximal Youden Index was 162.55 pg/mL, sensitivity was 83.8% and specificity was 98.9%, and AUC was 0.939. When it comes to the cut‐off value in clinic of 65.7 pg/mL, the sensitivity was 87.8% and specificity was 86.7%. And for NSE, the cut‐off value was 17.35 ug/L, sensitivity was 86.5% and specificity was 91.3% and AUC was 0.923. (Figure 2B). (Table 1).

### 3.3 Diagnostic efficiency of proGRP + NSE

Then, binary logistic regression of proGRP and NSE was conducted for joint application of the two biomarkers, and the resulted possibility was used for ROC curve. Consequently, when the two biomarkers were combined, the AUC and diagnostic sensitivity were higher than either of the results obtained from single biomarker, but the specificity lay between them. (Figure 2; Table 1).

### 3.4 ProGRP level and therapeutic response

**Figure 3:**
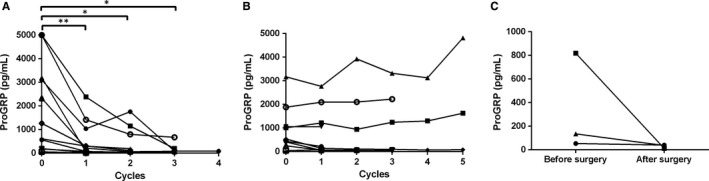
Change of proGRP levels before and after treatment. A, Change of proGRP levels of responders before and after chemotherapy; B, Change of proGRP levels of non‐responders before and after chemotherapy; C, Change of proGRP levels before and after surgery. proGRP, progastrin releasing peptide. *P < 0.05, **P < 0.01, ***P < 0.001

Change of proGRP levels before and after chemotherapy was analysed. For responders, plasma proGRP showed a downward trend after treatment. (Figure 3A) while for non‐responders, there was no obvious decrease in proGRP level before and after treatment (Figure 3B). Particularly, for responsive patients, proGRP level before the second cycle of treatment was significantly lower than that of baseline (before the first cycle of treatment), (*P* = .003); but in non‐responsive group, there was no significant decline in the concentration of proGRP (*P* = .173).

Five patients received surgery of pulmonary lesions, but only three patients had data of proGRP levels before and after surgery. For the first patient, proGRP level before surgery was 53.95 pg/mL, and which was 40.30 pg/mL after surgery; for the second patient, the proGRP level decreased dramatically after surgery when compared to preoperative level (818.80 pg/mL vs 11.82 pg/mL); and proGRP level of the third patient also reduced after surgery (135.20 pg/mL vs 36.78 pg/mL; Figure 3C).

**Figure 4:**
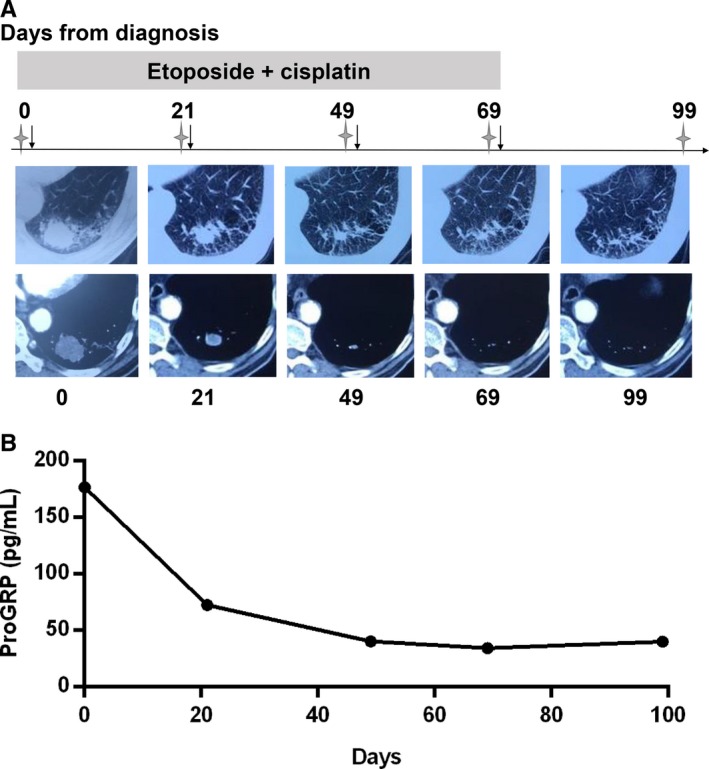
Dynamic change of imaging characteristics and proGRP levels of the patient who was responsive to treatment. A, imaging characteristics; B, proGRP levels. Stars, radiological examination was performed here (chest CT); Arrows, chemotherapy was given here. proGRP, progastrin releasing peptide; CT, computed tomography; MRI, magnetic resonance imaging

**Figure 5:**
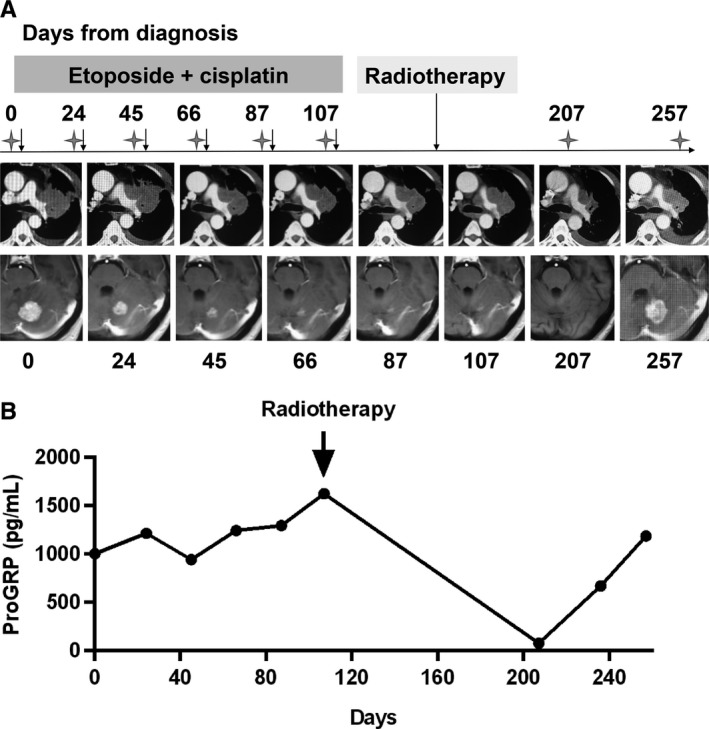
Dynamic change of imaging characteristics and proGRP levels of the patient who was unresponsive to treatment. A, imaging characteristics; B, proGRP levels. Note: After the fifth cycle of chemotherapy, only chest CT, but no cranial MRI, was performed; after the sixth cycle of chemotherapy, both chest CT and cranial MRI were not performed; stars, radiological examination was performed here (chest CT or cranial MRI); arrows, chemotherapy or radiotherapy was given here. proGRP, progastrin releasing peptide; CT, computed tomography; MRI, magnetic resonance imaging

To confirm if the change of proGRP levels was consistent with the state of illness, we explored the relationship between radiological characteristics of the solid tumour and proGRP levels. As is shown in Figure 4, the patient was diagnosed with limited stage SCLC and was treated with etoposide and cisplatin. Evidently, tumour reduction was obvious and the therapeutic response evaluation was PR. Synchronously, proGRP levels steady declined (Figure 4). However, there was another patient who was diagnosed as SCLC with brain metastasis. After 6 cycles of chemotherapy (etoposide + cisplatin), the pulmonary target lesion decreased less than 30% in the sum of diameters and the tumour response was assessed as SD; pleural effusion on the left side visually reduced; and intracranial metastasis shrunk drastically. Afterwards, radiotherapy of pulmonary and intracranial lesions was performed and we could see the pulmonary target lesion decreased dramatically; intracranial lesion even completely disappeared (Figure 5A). Interestingly, proGRP level was on the rise during chemotherapy. However, it dropped swiftly after radiotherapy (Figure 5B). However, at the last follow‐up visit, the disease progressed and so was the proGRP level elevated.

## 4 DISCUSSION

Our study showed that the diagnostic efficiency of proGRP on SCLC was superior to that of NSE. In 1990s, EGTM recommended NSE as the tumour biomarker assisting for the diagnosis of SCLC.^19^ However, up to 80% of NSCLC tissues could be stained with NSE, even if only 20%‐30% of the NSCLC patients had serum NSE level elevated.^20^ What's more, NSE elevation was present in many other malignant tumours as well as some benign lung diseases; the sensitivity of NSE on SCLC diagnosis was lower especially in diseases limited to one side of the thorax or the ipsilateral mediastinum^10^; and haemolytic samples must be removed because NSE exists in platelets and erythrocytes, and timely store of samples was crucial.^20^ Meanwhile, several studies demonstrated proGRP is more precise in therapeutic monitoring, recurrence predicting and prognosis forecasting in limited stage SCLC.^18^, ^21^, ^22^, ^23^, ^24^ In benign diseases, renal insufficiency is an important factor which may lead to proGRP level increase. Slight elevation of proGRP level may be visible in many other malignant tumours, but 99.7% of them <100 pg/mL.^25^ It is now proved that proGRP could be a reliable biomarker of SCLC patients, and it was superior to NSE on sensitivity, specificity and reliability.^10^, ^13^, ^14^, ^22^, ^26^ Therefore, more and more studies recommended proGRP as the biomarker for SCLC diagnosis because of the high sensitivity and specificity; early elevation; normal in most diseases except of renal dysfunction; no false positive because of haemolysis; and the marked differences between SCLC patients and normal populations. Particularly, NSE could be the complementary biomarker. Combined application of NSE and proGRP could increase the accuracy of histologic diagnosis, prognosis and follow‐up visit.^27^

Proper selection of the optimal cut‐off value is essential. In our results, when discriminating SCLC from NSCLC, the cut‐off value was 114.35 pg/mL, while for SCLC and BLD, the cut‐off value was 162.55 pg/mL. However, the cut‐off value recommended with the kits is 65.7 pg/mL, which improves diagnostic sensitivity slightly but cut down the specificity dramatically. Apparently, the higher the cut‐off value, the lower the sensitivity and the higher the specificity, vice versa. From a clinical point of view, early diagnosis of SCLC would benefit patients more and prolong survival, thus higher sensitivity takes precedence over specificity. Anyhow, proGRP is an auxiliary test and it must be combined with clinical characteristics and the results of other examinations for the diagnosis of SCLC, especially histopathological findings.

In our study, there was no significant difference of proGRP levels before the first cycle of treatment between responders and non‐responders. However, marked difference between them was observed before the second cycle of treatment. Just as Holdenrieder et al demonstrated that high levels of proGRP before the first cycle of treatment, as well as the insufficient decline in proGRP level before the second cycle of treatment were related to the poor outcome of SCLC patients; and proGRP level before the second cycle of chemotherapy was the early estimation of therapy response, reaching the AUC of 71.3% in ROC curve. All these results suggested that for responsive patients, distinct decrease in proGRP levels could be visible even after once of treatment; otherwise, it may be predictive of poor response to treatment. Particularly, the proGRP level rose in volatility during chemotherapy when pulmonary focus was evaluated as SD, despite of the visual reduction of intracranial lesion. However, it dropped swiftly after radiotherapy when the pulmonary target lesion decreased markedly, which indicated that proGRP could better reflect the therapeutic response of pulmonary target lesions, other than metastasis lesions. However, only three patients with surgery were reported here and further study should be conducted to investigate how proGRP levels change before and after surgery.

The method used in our study for measurement of proGRP level was Elecsys ProGRP assay, which could be traced to the ARCHITECT of Abbott Diagnostics. For ARCHITECT assay, plasma samples were recommended because proGRP was less stable in serum. More than 50% of serum proGRP would be degraded after 72 hours in room temperature, but stored by deep freezing could markedly inhibit the degradation.^28^ The reason for less stability in serum may be thrombin induced protein hydrolysis. Elecsys ProGRP assay uses the monoclonal antibodies that targeted the 48‐52 and 57‐61 amino acids of proGRP, which avoids the cutting site of thrombin and as a result, both plasma and serum could be used for assay.

We recognized some limitations of this study. Firstly, it was the preliminary results of a single centred retrospective study. Additionally, no patients with other malignant tumours were enrolled and analysed. Besides, the upper limit of proGRP level measured by Elecsys ProGRP Assay was 5000.00 pg/mL, and non‐parametric analysis was adopted to weaken their impact on results. Furthermore, no information of survival and prognosis was analysed in our study and further study is needed to explore whether the change of proGRP level could predict the long‐term effect of treatment, such as disease‐free survival and overall survival.

In conclusion, proGRP is more precise for SCLC diagnosis when compared to NSE, and it could be a very valuable biomarkers for therapeutic predicting. But further multicentre prospective study with large samples was needed to validate current findings.

## CONFLICT OF INTEREST

The authors declare that they have no conflict of interest statement.
